# Taste of breath: the temporal order of taste and smell synchronized with breathing as a determinant for taste and olfactory integration

**DOI:** 10.1038/s41598-017-07285-7

**Published:** 2017-08-21

**Authors:** Yuya Kakutani, Takuji Narumi, Tatsu Kobayakawa, Takayuki Kawai, Yuko Kusakabe, Satomi Kunieda, Yuji Wada

**Affiliations:** 1 BKC Research Organization of Social Sciences, Ritsumeikan University, 1-1-1 Noji-higashi, Kusatsu, Shiga 525-8577 Japan; 2 Laboratory of Sensory Science, Food Research Institute, National Agriculture and Food Research Organization, 2-1-12, Kannondai, Tsukuba, Ibaraki 305-8642 Japan; 3 Graduate School of Information Science and Technology, the University of Tokyo, 7-3-1, Hongo, Bunkyo-ku, Tokyo 113-8656 Japan; 4 Human Technology Research Inst., National Institute of Advanced Industrial Science and Technology (AIST), Tsukuba Central 6, 1-1-1 Higashi, Tsukuba Ibaraki, 305-8566 Japan; 5 Advanced Materials & Technology Research, Corporate Research & Development Division, Takasago International Corporation, 1-4-11 Nishiyawata, Hiratsuka, Kanagawa 254-0073 Japan; 6 College of Science and Engineering, Ritsumeikan University, 1-1-1 Noji-higashi, Kusatsu, Shiga 525-8577 Japan

## Abstract

Many studies have reported that subjective taste intensity is enhanced by odors which are congruent, for example a sweet taste and a vanilla odor. Some reports have suggested that subjective taste is more strongly enhanced by retronasal than by orthonasal odors; others have suggested that taste enhancements by both odor routes are identical. Differences between the two routes include the direction of airflow accompanying breath. Thus, it is possible that the order of gustatory and olfactory stimuli when breathing through either route while drinking is a determining factor for taste-odor integration. To reveal the natural relationship between taste intensity enhancement by odors and breath, synchronization of odor stimulation with the breath is necessary. Here, we examined whether the enhancement of a sweet taste is induced by a vanilla odor presented in various combinations of odor routes, immediately before and immediately after drinking. The results showed that a retronasal odor after drinking enhanced taste, but an orthonasal odor before drinking did not. The retronasal odor before drinking and the orthonasal odor after drinking did not enhance the sweet taste. These results show that congruency with the natural order of stimulus and kinetic sensation is a determining factor for odor-induced taste enhancement.

## Introduction

When we eat or drink, we experience food and beverage not only through our so-called sense of taste, but also through smell and other inputs^1^. Humans create percepts of food and beverages based on information gleaned through sensory modalities including taste and smell^1, 2^.

Taste and smell are closely related in our perception. Odor molecules reach the olfactory epithelium by two routes. One is the orthonasal route, which is through the nose, and the other is the retronasal route, which is through the mouth^3, 4^. Rozin asserted that orthonasal odors tend to be localized to the external world and retronasal odors tend to be localized to the oral cavity, leading humans to mistake retronasal olfaction for “taste”^3^. Recently, Linscott and Lim (2016) reported that the effect of odor on taste enhancement was a consequence of the halo-dumping effect^5^. The halo-dumping effect is a bias due to the omission of potentially salient rating scales. For example, when subjects are provided with only one rating option, such as sweetness, rather than all appropriate rating categories, they dump their odor intensity rating onto their taste intensity rating^6^.

It has been suggested that the same olfactory stimulation may be perceived and evaluated in two qualitatively different ways, depending on whether it is referred to as coming from the mouth or from the external world^3, 7, 8^. This localization bias is strongly affected by the congruency between olfactory and gustatory stimuli^9–11^. In addition, it was reported that subjects who wore a nose clip before, during and after eating a vegetable stimulus rated the intensity of tastes such as sweetness, bitterness and saltiness as weaker than usual^12^. This phenomenon occurs because airflow with odor molecules from the mouth to the nose is disrupted and blocks flavor perception. In other words, both smell and taste are strongly related to our everyday flavor or taste perceptions. Many studies have examined the interaction between odor and taste. For instance, associations between a strawberry odor and sweetness^13, 14^, caramel and sweetness^15^, waterchestnut and sweetness^16^, ethyl hexanoate (sweet smelling) and sweetness^17^, dried bonito stock and umami^18^, and soy sauce and saltiness^14^ have been reported. In these studies, the odor stimuli were mixed in a drink containing gustatory stimuli. Strawberry, caramel and vanilla odors tend to enhance the sweetness of a sucrose solution^15, 19^.

As noted above, many phenomena related to taste-odor interaction have been reported upon, but few studies have focused on the effects of differences in the route of odor delivery, which closely relate to breath in daily life. Some reports have found that odor-induced taste enhancement occurred not only when gaseous odor stimuli were presented via the retronasal route, but also via the orthonasal route^14, 20^. While these findings suggest that odors presented by either route enhance taste perceptions, it remains unknown whether or not the effects of both routes on taste intensity are identical. In addition, the binding of taste and smell by mislocalizing odors is influenced by relative stimulus timing^9^. Recently, Isogai *et al*. conducted an experiment to determine whether modulation of taste by retronasal odor is dependent on temporal contiguity^17^. In this experiment, onset of odor presentation ranged from 2 s before taste delivery onset to 2 s after taste delivery onset in 1-s increments^17^. Their results showed that enhancement of taste intensity was greatest with simultaneous onset of taste and odor^17^. While these findings suggest that temporal synchrony is important for odor-induced taste enhancement, it remains unknown whether the temporal order of smells from both routes impacts taste.

It is to be noted that the differences between orthonasal and retronasal routes include the direction of airflow accompanying breath since odor molecules along these routes are received by olfactory receptors in the same olfactory epithelium^8^. Here, we can assume that one significant cue for the perceptual distinction between orthonasal and retronasal routes might be the motor sensation of breath. Which is to say that with natural eating and drinking, orthonasal and retronasal odors could be perceived while breathing in before intake and while breathing out after intake, respectively. It is possible that the order of olfactory stimulation (via either route) and intake, together combined with breath, is related to taste-odor integration or the halo-dumping effect. In order to reveal the natural relationship between taste intensity enhancement by odors and breath, synchronization of odor stimulation with breath is necessary.

To examine this hypothesis, we compared the intensity of odor-induced taste enhancement where the temporal order of taste and smell stimuli is congruent with daily beverage intake and that where the temporal order of taste and smell stimuli is incongruent by using an olfactory display we developed.

## Experiment 1

In Experiment 1, we examined whether the odor route (orthonasal or retronasal) changes the odor-induced taste enhancement caused by the odorant in the air reaching the olfactory epithelium accompanying breath. In order to examine this issue, we developed an olfactory delivery unit and display system which allowed the presentation of odor stimuli by both routes in synchrony with participants’ breath. The intensity of smells under the respective odorant route conditions must be the same in order to compare the effect of route difference on taste. Thus, we excluded data for participants who exhibited a significant difference in perceived odor intensity between orthonasal and retronasal conditions during a screening session.

### Materials and Methods

#### Participants

Nine healthy females and five healthy males were recruited from the National Food Research Institute. Participants were instructed not to eat or drink anything except water for at least one hour before the experiment. They self-reported being neither hungry nor full, and they had no olfactory or gustatory deficits and no health problems.

The experimental protocol was approved by the ethical committees of the Food Research Institute, the National Agriculture and Food Research Organization and of the University of Tokyo. The participants gave written informed consent. The study was conducted in accordance with the Declaration of Helsinki.

#### Apparatus

**Figure 1:**
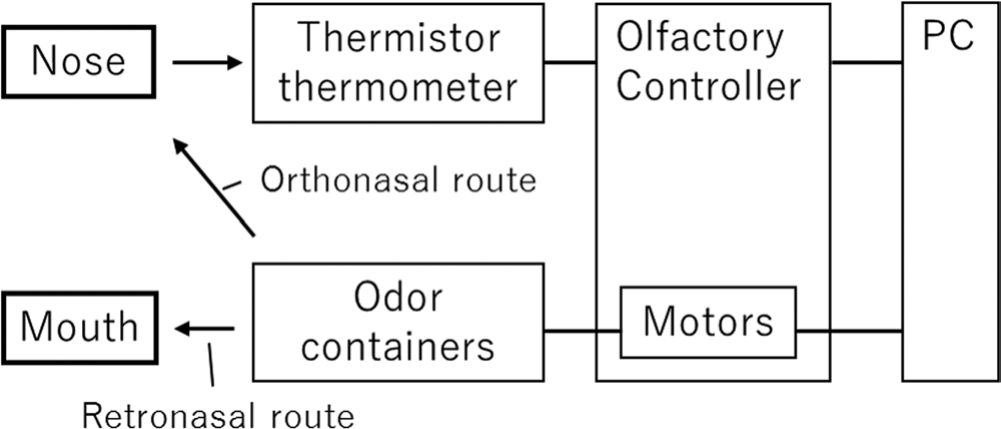
System configuration of the device.

Based on the Meta Cookie system, which incorporates a display and olfactory stimuli delivery system using air pumps controlled by a computer^21^, we developed an olfactory delivery system and display that can present olfactory stimuli synchronized with breathing via both routes of odor delivery^22^ (Fig. 1).

The olfactory delivery system and display were controlled using a personal computer (ENVY 700-270jp, HP Japan Inc., Tokyo, Japan). The delivery system had built-in motor pumps connected to containers that contained either an odorant or water. Each odor container had two glass tubes. One of the glass tubes was for taking in air from the motor pump, and the other was for delivering air with an odorant or with water to participants via a polytetrafluoroethylene tube on a headset. The headset had two tubes to present airflow to the nasal cavity and the oral cavity, respectively. One tube was inserted about 5 mm into the nostril in order to present the odor via the orthonasal route, and the other was connected to a bendable straw which each participant positioned near their throat in order to present the odor via the retronasal route.

In order to avoid participants detecting stimulus presentation by the change in airflow pressure accompanying the odor, airflow was maintained through both tubes during each trial at a stable flow rate of 1.15 l/min. Odor presentation could be switched between the pumps connected to the container containing the odorant and that containing water. In a previous test, we measured the temporal accuracy of pump switching for this device in order to confirm the accuracy of olfactory stimulation by using a high-speed gas concentration monitoring device^23^. For this device, the time lag between the switching of the visual display and olfactory controlled by the PC was about 0.21 s (SD = 0.02)^22^, which is short enough to present the odor stimulus with an inhalation or exhalation that lasts about 1 s detected by the sensor.

In addition, white noise was presented from the headset during the trials to mask the motor sound.

The olfactory controller controlled the air pumps according to the state of breath as measured by a thermistor thermometer positioned in front of the nostril. We used a visual monitor (LCD-AD173CWR, I-O DATA DEVICE Inc., Ishikawa, Japan) to instruct participants in the timing of breathing and drinking. Participants’ breathing was monitored with the thermistor thermometer to synchronize olfactory stimuli with breathing and to control the total time of presentation for each trial.

In our previous study, we tested whether the intensity of a vanilla odorant stimulus delivered by the device could be respectively perceived via both orthonasal and retronasal routes^22^. We delivered the odor through the nose as participants breathed in, and through the mouth as they breathed out using this device. Participants rated the intensities of stimuli using visual analogue scales. The results showed that participants were able to rate odor intensity in accordance with odorant concentration via both routes^22^.

#### Stimuli

Vanilla essence (Kyoritsu Foods Co. Inc., Tokyo, Japan) was used as the olfactory stimulus because it is well known that a vanilla odor enhances sweet tastes. Cotton placed into heat-resistant plastic bottles was permeated with the olfactory stimulus. To supply the olfactory stimulation efficiently, the bottles were heated to 65 °C on a hot stirrer plate during the experiment. In our previous study^22^, the difference in perceived intensity between odor stimuli delivered via the orthonasal route and the retronasal route was not significant. In addition, we conducted a pilot experiment to confirm that the odor stimulus used in this study would not stimulate the taste receptors directly. Three participants with their noses pinched closed with a clip in order to block the retronasal olfactory stimulation were presented odorless air or odor (the same vanilla as used in Experiment 1) stimuli to the oral cavity in random order. No participants perceived any taste with either the odorless air or the odor stimuli. These results reveal that the odor stimuli used in the experiment did not induce any taste perception when they did not stimulate the olfactory receptor.

The sucrose solutions for drinking were prepared using sucrose (granulated sugar) and pure water. We used 2 stimuli solutions: 0.0 and 2.0% weight for weight (w/w). In addition, a 3.0% w/w solution was used as a reference stimulus. The solutions were served at room temperature.

#### Procedure

Each participant wore an olfactory-delivery-system headset and was seated in front of a computer monitor (display). They took part in a three-part screening test: training, taste evaluation and smell evaluation.

**Figure 2:**
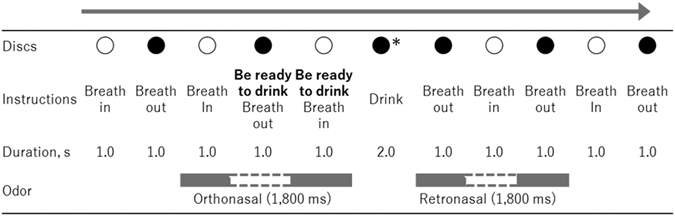
Time course of instruction and discs in taste evaluation phase (Experiment 1). The odor stimulus was presented for a combined maximum of 1.8 s during two successive inhalations or two successive exhalations. The grey bars represent odor delivery; the white gaps represent a pause in odor delivery. *In the actual experiment, we displayed a red disc when participants were instructed to drink the solution.

**Figure 3:**
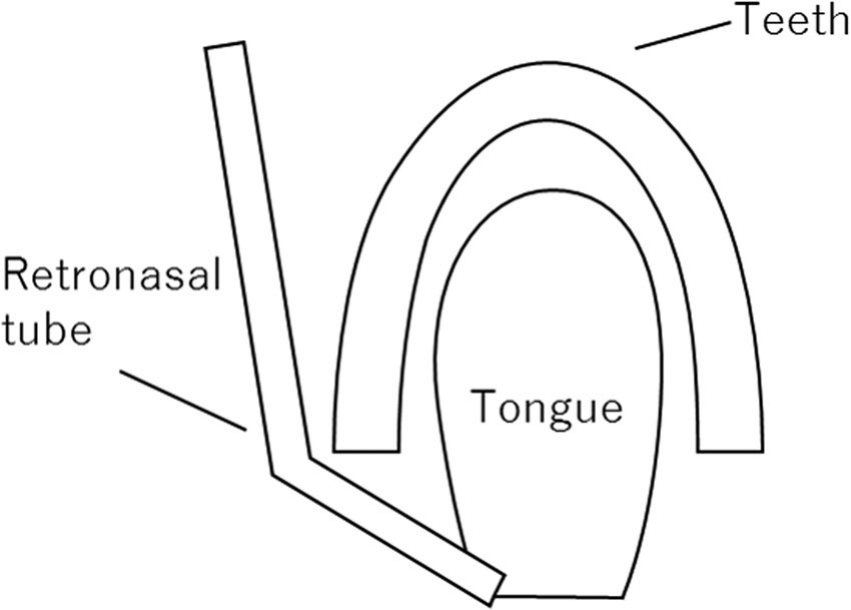
Illustration of placement of tube for retronasal stimulation.

At the beginning of the experiment, participants were given a few minutes to adapt to breathing in synchrony with instructions on the display while airflows were introduced into the nose and mouth via tubes. A series of white (inhale), black (exhale) and red (drink) discs and instructions were presented on the display to present the breathing rhythm and drink timing. Since the displayed instructions and symbols were changed every 1,000 ms, one full breath (inhalation and exhalation) was completed in 2 seconds (Fig. 2). The participants were instructed to bite the straw with the left retromolar area to fix the end of the straw near their throat as in Fig. 3, and to close their mouth and breathe through their nose. After the breath synchronization phase, participants practiced drinking the water solutions as gustatory stimuli while breathing in synchrony with the instructions on the visual monitor. Participants were asked to rinse their mouths with water after each tasting. Each participant inserted the end of a tube attached to a syringe into his or her mouth at the onset of the tasting period. Figure 2 shows the time course of stimulation for each trial. The participants injected the gustation solution into their oral cavity in one quick shot (one-shot drinking) by themselves in accordance with the instructions on the display. We changed the syringe for each trial in order to avoid mixing tastes. During each trial, approximately 5 ml of the gustatory stimulus was delivered. When instructions and a red disc, which was the sign to drink, were displayed for 2 seconds, the air pumps were stopped because airflow interfered with drinking, and participants injected the solution by themselves and drank it. After swallowing, the participants continued breathing in accordance with the instructions on the display. After the stimulus was presented, participants were instructed to rate the intensity of tastants while odorants were presented via orthonasal or retronasal routes.

The taste evaluation part of the experiment consisted of 3 sessions. Each session included 6 trials of a two-factorial design with 3 odor conditions (odorless, orthonasal, or retronasal) and 2 solution conditions (0.0 and 2.0%). Participants were not informed whether the odorants were delivered nor by which route during each trial, nor were they told how many and what kinds of solutions and odor stimuli were being used until all experiments were finished. As with the training phase, the participants were instructed to inject and drink a water solution while breathing in synchrony with the instructions on the display. The olfactory stimuli were delivered into the nose (orthonasal) or the mouth (retronasal) automatically and synchronously with breathing via a polytetrafluoroethylene tube attached to pumps on the olfactory unit. The odor stimulus was presented for a combined maximum of 1.8 s during two successive inhalations or two successive exhalations, depending on the trial condition. As noted above, the delivery system was sensitive enough to pause the odor stimulus for the exhalation or inhalation between the two target breaths (see Fig. 2). Odors delivered to orthonasal or retronasal routes were presented during the inhalation before drinking and during the exhalation after drinking, respectively, to reproduce a natural drinking-breathing order (Fig. 2). At the end of each trial, participants rated the strength of taste (sweetness of the sucrose solution) using a visual analog scale on a laptop PC (CF-AX3, Panasonic Corporation, Osaka, Japan). This scale was a 160-mm line scale labelled “no taste” (0 mm) at the left end, “extremely strong taste” (160 mm) at the right end and “reference taste” (80 mm) at the middle. The reference solution (3.0% sucrose) was presented prior to each session. Participants were instructed that this reference corresponded to the middle of the scale. Participants were asked to thoroughly rinse their mouth with water after each trial. The time interval between samples was approximately 100 s. As 6 stimuli were presented for each session in random order, the participants performed 18 trials.

To confirm each participant’s olfactory performance, a smell evaluation was conducted as part of the screening test. The procedure was almost identical to that for the rating of taste intensity, with only the rating target being different. The participants provided ratings for the odor stimulus intensity after each trial, using a visual analog scale on a laptop PC. This scale was a 160-mm line scale labelled “no odor” (0 mm) at the left end, “extremely strong” (160 mm) at the right end and “reference odor” (80 mm) at the middle. The reference stimulus presented at the beginning of the session was identical to the olfactory stimulus in the taste evaluation phase.

#### Data analysis

Data analysis was performed using R version 3.2.0 (R Core Team, 2015). To screen out the participants who experienced a significant difference in odor intensity between olfactory routes, paired t-tests were performed on each participant’s ratings of odor intensities for orthonasal and retronasal routes provided during the smell evaluation phase.

For each participant, we calculated the mean ratings across all conditions in order to examine whether the odor stimulus enhanced taste intensity, mean ratings for taste intensity were calculated for each trial within-participants. A 2 * 3 (taste condition * odor condition) repeated measures analysis of variance (ANOVA) was performed on the mean rates of taste intensity. If a significant factor was determined with ANOVA, post hoc comparisons between independent variables were conducted using Ryan’s method. All reported p-values were two-tailed, and values of <0.05 were considered to be statistically significant.

### Results

As a result of the screening test, four participants were excluded from the analysis because they experienced significantly different intensities in the odors delivered to the retronasal and orthonasal routes. The final sample consisted of ten participants, including six females and four males (mean age = 39.4 years, SD = 6.2, range: 28–47).

**Figure 4:**
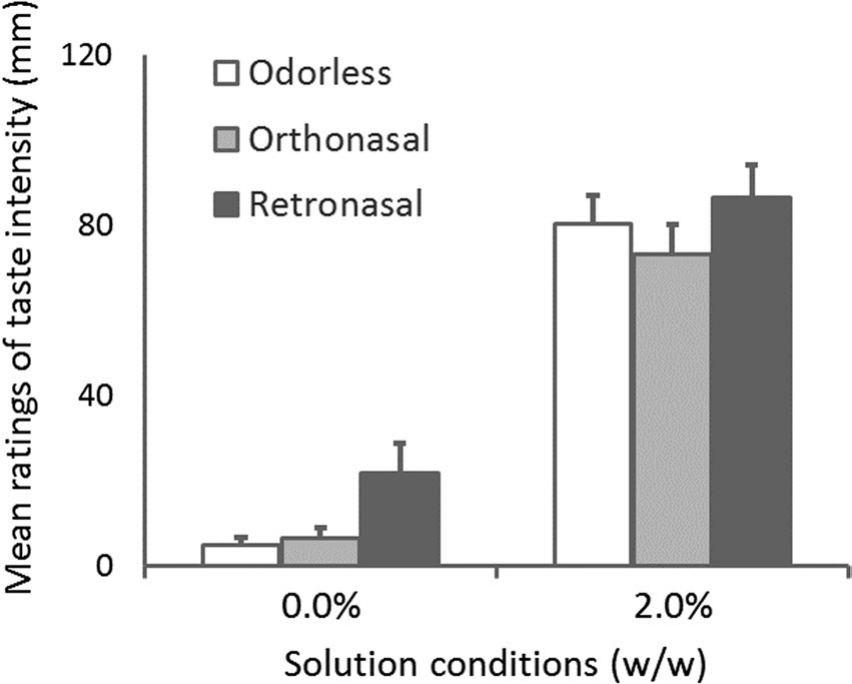
Mean ratings of taste intensity for Experiment 1. Error bars indicate standard error (n = 10). A two-way ANOVA showed significant main effects of solution (F (1, 9) = 53.2, p = 0.00005, η_p_^2^ = 0.86) and odor (F (2, 18) = 8.1, p = 0.003, η_p_^2^ = 0.47), but the interaction between factors was not significant. Post hoc analysis showed that the ratings of taste intensity for the retronasal route condition were significantly higher than those for the orthonasal route condition (p = 0.025, η^2^ = 0.58) and were marginally higher than those for the orthonasal route condition and odorless condition (p = 0.018, η^2^ = 0.59: p = 0.048, η^2^ = 0.42).

A 2-way ANOVA was conducted for the mean ratings of taste intensity in Experiment 1, with the two factors being odor condition (odorless, orthonasal, or retronasal) and solution condition (0.0 or 2.0%). Figure 4 shows the mean rate for intensity of taste.

We found main effects of solution (F (1, 9) = 53.2, p = 0.00005, η_p_^2^ = 0.86) and odor (F (2, 18) = 8.1, p = 0.003, η_p_^2^ = 0.47), but the interaction between factors was not significant. Post hoc analysis showed that the rates of taste intensity for the retronasal route condition were significantly higher than those for the orthonasal route condition and odorless condition (p = 0.018, η^2^ = 0.59: p = 0.048, η^2^ = 0.42).

### Discussion

The results of Experiment 1, in which olfactory stimuli were separated from gustatory stimuli and presented synchronously with breathing in, demonstrate that a retronasal vanilla odor after one-shot drinking enhanced a sweet taste, but an orthonasal vanilla odor before one-shot drinking did not.

The main effect of solution revealed with a 2-way ANOVA indicates that participants’ ratings of the intensities of the tastes corresponded with the sucrose concentrations of the solutions. We did not find significant differences between the odorless condition and the orthonasal condition, suggesting that the orthonasal vanilla odor presented before drinking did not enhance the sweet taste of the solutions. In contrast, taste intensity under the retronasal odor condition was stronger than that under the odorless and orthonasal condition. These results suggest that a retronasal odor after drinking, which is congruent with a natural drinking pattern, enhances perceived taste intensity.

However, we cannot identify whether the retronasal route, the timing of the odor stimulus relative to swallowing the gustatory stimulus or both is the determining factor for taste enhancement because order was a confounder in this design. Thus, in order to clarify whether or not the relationship between natural breath and taste intensity enhancement by odors is the determining factor for odor-induced taste enhancement, in a second experiment (Experiment 2), we examined whether a retronasal odor presented before drinking and an orthonasal odor presented after drinking enhance taste intensity.

## Experiment 2

In Experiment 1, we found that a retronasal vanilla odor presented after drinking enhanced a sweet taste, but that an orthonasal vanilla odor presented before drinking did not. However, it was impossible to distinguish the effect of odor route from that of odor timing relative to gustatory stimulation because order was a confounder. In Experiment 2, therefore, we examined whether the taste enhancement by odor is observed when the order of the odor stimulus routes (orthonasal and retronasal) and solution was flipped. If only the difference of odor routes (relative timing of smell) was a determining factor for the taste enhancement observed in Experiment 1, we should observe taste enhancement under the retronasal (orthonasal in Experiment 1) condition. If the combination of retronasal odor and the timing of stimulation is a determining factor for taste enhancement by odor, we should not observe an effect of odor route.

### Materials and Methods

#### Participants

Eight healthy females and six healthy males were recruited from the National Food Research Institute. The participants were instructed not to eat or drink anything except water for at least one hour before the experiment. They self-reported being neither hungry nor full, and they had no olfactory or gustatory deficits and no health problems. To confirm the participants’ olfactory performance, an odorant-rating session was performed.

The experimental protocol was approved by the ethical committees of the Food Research Institute, the National Agriculture and Food Research Organization and of the University of Tokyo. The participants gave written informed consent. The study was conducted in accordance with the Declaration of Helsinki.

#### Apparatus and stimuli

The device, odorants and tastants used in this experiment were the same as those of Experiment 1.

#### Procedure

**Figure 5:**
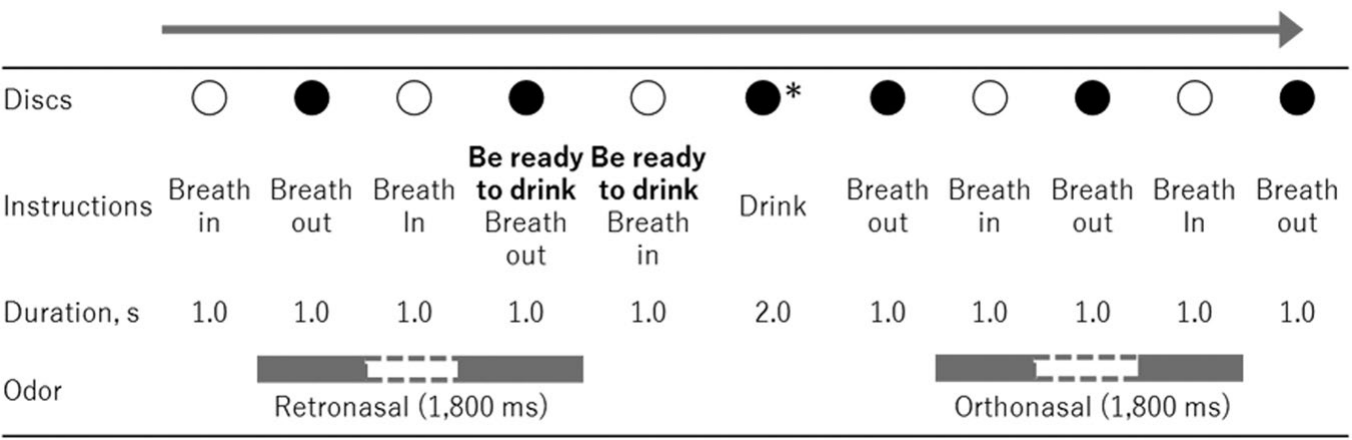
Time course of instruction and discs for the taste evaluation phase (Experiment 2). The odor stimulus was presented for a combined maximum of 1.8 s during two successive inhalations or two successive exhalations. The grey bars represent odor delivery; the white gaps represent a pause in odor delivery. *In the actual experiment, we displayed a red disc when participants were instructed to drink the solution.

The procedure was almost identical to that of Experiment 1, with only the timing of odor presentation differing. In Experiment 2, the vanilla odor introduced via the orthonasal route and that via the retronasal route were presented during the inhalation after presentation of the taste stimulus and during the exhalation before the taste stimulus, respectively (Fig. 5).

#### Data analysis

The data analysis was as that of Experiment 1.

### Results

As a result of the screening test, six participants were excluded from the analysis because their ratings of the strengths of odors from the retronasal and orthonasal routes were significantly different. The final sample was eight participants, including four females and four males (mean age = 36.9 years, SD = 6.9, range: 25–47). Six of the participants in Experiment 2 were the same as those in Experiment 1.

**Figure 6:**
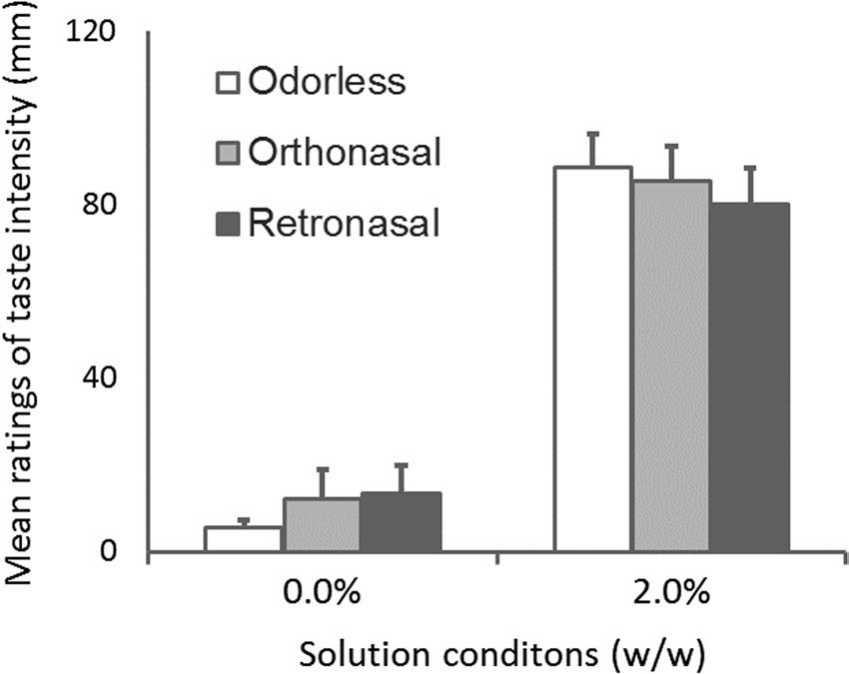
Mean ratings of taste intensity for Experiment 2. Error bars indicate standard error (n = 8). A two-way ANOVA showed that the main effect of solution was significant (F (1, 7) = 293.3, p = 0.0000006, η_p_^2^ = 0.98), but the main effect of odor and the interaction between factors were not significant.

A 2-way ANOVA was conducted for the mean ratings of the tastants in Experiment 2, with the two factors being odor condition (odorless, orthonasal, or retronasal) and solution condition (0.0 or 2.0%). Figure 6 shows the mean ratings for taste.

We found a main effect of solution (F (1, 7) = 293.3, p = 0.0000006, η_p_^2^ = 0.98), but the main effect of odor and the interaction between factors were not significant.

### Discussion

The results of Experiment 2, in which olfactory stimuli were separated from gustatory stimuli and presented synchronously with breathing in, reveal that a retronasal vanilla odor presented before one-shot drinking and an orthonasal vanilla odor presented after drinking did not enhance the sweet taste.

The main effect of solution indicates that participants’ ratings of the intensity of tastes corresponded with the sucrose concentrations of the solutions. We did not find significant differences between the odorless, orthonasal and retronasal conditions, which suggests that an orthonasal vanilla odor presented after one-shot drinking and a retronasal vanilla odor presented before drinking did not enhanced the sweet taste of the solutions. These results suggest that an orthonasal odor presented after drinking and a retronasal odor presented before drinking, which is incongruent with a natural drinking pattern, does not enhance perceived taste intensity.

## General Discussion

The primary goal of these experiments was to determine whether the relative order of taste and smell aligned with inhalations and exhalations is associated with orthonasal and retronasal routes. Our results indicate that taste enhancement is induced by odor from the retronasal route after one-shot drinking, but not induced by odor from the retronasal route before one-shot drinking: the perceived sweetness of a sucrose solution was enhanced by the vanilla odor when presented via the retronasal route after drinking. In addition, this enhancement was not observed in Experiment 2, in which the timing of the odor presentation was reversed.

Flavor perception is known to be influenced by information cues provided before tasting^24, 25^. However this was not the case in the present study, because participants were not forewarned about which stimuli they would experience and the stimuli were presented in random order for each session. It must be noted that it is possible that the halo-dumping effect affected the taste enhancement observed in this study: the vanilla odor we used is well known as a sweet smell, and participants were provided only with rating options for sweetness. Thus, it remains unclear whether the current findings are due to perceptual or cognitive processing. However, the results of this study cannot be explained by the halo-dumping effects alone. In Experiment 2 of this study, although only the order in which odor and taste were presented was switched compared with Experiment 1, odor-induced taste enhancement was eliminated. Therefore, it is not plausible that the taste enhancement observed in Experiment 1 is simply due to the effects of halo dumping. Further research should examine whether taste enhancement by odor as observed in our experiment is caused by the halo-dumping effect or the integration of gustation and olfaction. In either case, it is clear that a retronasal odor after drinking enhances the subjective intensity of taste.

Odor stimuli delivered via the retronasal route were introduced into the oral cavity using a tube. Therefore, it may be observed that the odor stimuli in this experiment stimulated not only the olfactory epithelium, but also somatic sensation^8^. There is a possibility that the interaction between the trigeminal nerve and the olfactory system relates to the differential perception of orthonasal and retronasal stimuli^26^. However, the oral cavity trigeminal system is fully unresponsive to purely olfactory stimuli such as vanillin^27^. Furthermore, we can ignore somatosensory stimulation as a determining factor for the taste enhancement we observed, as participants could not distinguish between orthonasal and retronasal delivery of odorless air because there was no change in airflow for either route during each trial.

We can ignore the possibility that the vanilla odor stimulated taste because in a pilot study, we confirmed that no participants perceived any taste with the odor stimuli used in our experiments. The results of the pilot study eliminate the possibility that the vanilla odor became a taste stimulant perceivable by the participants as result of interaction with saliva. Indeed, since taste enhancement did not occur when the time order of gustatory and olfactory stimuli was incongruent, it is more likely that the relationship with breathing impacted the taste enhancement.

Since Rozin’s hypothesis, some studies have confirmed that the same olfactory stimulation can be perceived and evaluated in two qualitatively different ways depending on route^7, 28, 29^. Using an olfactory display to differentiate both routes, our results support this idea. On the other hand, some reports have suggested that there is little functional difference between retronasal and orthonasal olfaction^20^. Sakai and colleagues suggested that there is no obvious functional difference between orthonasal and retronasal olfaction^20^. A possible reason for this inconsistency might be a difference in the intensity of the stimuli. In the current study, odor stimuli were presented by a slow airflow, and only during the inhalation or the exhalation. In the study by Sakai and colleagues, odor stimuli were consecutively presented to the oral or nasal cavities for durations of 400 ms^20^. The odor stimuli were presented only before or after the taste stimulus in the current study. In addition, in our study, the total duration of the odor stimulus was controlled. In contrast, the odor stimuli were consecutively presented in the study by Sakai and colleagues^20^. Thus, we can expect that the intensity of the odor stimuli in our experiments might be weaker than that in previous studies, and thus possibly too weak to enhance the sweet taste under some conditions.

Our olfactory delivery system and visual display allowed us to synchronize the olfactory stimulation with breath via both routes and to make apparent the difference between the effect of orthonasal and retronasal odors on taste. Since differences in airflow patterns are thought to contribute to perceptual differences between orthonasal and retronasal presentation of odors^30^, synchronization with breathing is essential to evaluate differences in taste enhancement effected by route. Nevertheless, there are almost no previous studies in which odor is presented synchronously with breathing, making the current study novel. The results of the current study reveal that taste enhancement is induced by odors presented via the retronasal route, but not induced by odors presented via the orthonasal route. Thus, it is suggested that there is a cognitive difference between orthonasal and retronasal routes when an odor is presented synchronously with breathing.

Furthermore, in our experiments, retronasal-odor-induced taste enhancement was not observed when the vanilla odor was presented before the taste stimulus. On the other hand, when the odor was presented after taste, only the retronasal odor enhanced taste intensity, the orthonasal odor did not. Here we can conclude that not only the odor route, but also the temporal relationship between odor and taste were required to induce the taste enhancement. We can assume that one significant cue for the distinction between orthonasal and retronasal routes may only be the direction of airflow accompanying breath since odor molecules from both routes are received by olfactory receptors in the same olfactory epithelium^8^. The odor timed with the exhalation after taste in Experiment 1 is analogous to natural odor and taste timing whereas the odor timed with the exhalation before taste in Experiment 2 is incongruent. Thus, the timing congruency with natural tasting and kinetic sensation might be a determining factor for odor-induced taste enhancement. Some previous studies have reported that odor presentation induces taste enhancement using odor stimuli dissolved in the taste stimuli^13, 15, 16^. Here we developed a device that controls odor presentation in accordance with the timing of ingestion, and shows the relationship between the timing of odor presentation and taste enhancement. In the current study, to examine odor-induced taste enhancement, sucrose and vanilla were used as gustatory and olfactory stimuli, respectively. Moreover, we evaluated taste enhancement under controlled conditions rather than with natural drinking. Future studies that evaluate whether a similar phenomenon occurs with the combination of different stimulants and with natural drinking are needed. However, to the best of our knowledge, the current study is the first study reporting an enhancement of perceived sweetness induced by odor presented synchronously with breathing after drinking, and may contribute to the elucidation of the influence of odor presentation timing on taste enhancement.

In our results, the effect size of taste enhancement by odor condition was smaller than that by solution condition (odor: η_p_^2^ = 0.47, solution: η_p_^2^ = 0.86). There are some possible explanations for the differences between the results of previous studies and those of the current study. One possibility is that the odor intensity here might be weaker than that of previous studies. The airflow was stable at 1.15 l/min in order to avoid inflicting discomfort on participants who were exposed to airflow to both the nose and the oral cavity. This weak airflow might contribute to a reduction in the intensity of odor stimuli. Another possibility is that the continuity of odor was insufficient to enhance taste because we controlled odor presentation using a breath sensor. Since the odor stimulus was presented for a combined maximum of 1.8 s over the course of either two successive inhalations or two successive exhalations, participants were exposed to the odor for a very short time. This short exposure might depress odor-taste congruency. In either case, it is clear that a retronasal vanilla odor presented after a sweet taste enhanced the intensity of perceived sweetness more strongly than in other conditions because we strictly controlled stimuli and breath under all experimental conditions.

In this study, the presented odor concentration could not be stringently controlled because the bendable straw used for retronasal odor presentation was positioned near the throat by the participant her/himself. We could not fully avoid individual differences in straw position in the oral cavity and in sensitivity for odor from both routes. However, a pilot study confirmed that the intensity of the vanilla odorant stimuli delivered by this device could be respectively perceived via both orthonasal and retronasal routes^22^. In order to enable the perception of odor via both orthonasal and retronasal routes, we used an undiluted odor, which is commercially available, in the current study. In addition, we conducted a screening test for smell evaluation in order to exclude the data of participants who experienced significant differences between orthonasal and retronasal smell sensitivity. Therefore, we can assume that difference between the odor intensity for orthonasal stimulation and that for retronasal stimulation can be ignored in this study.

Finally, we must note that the order of the odor from both routes and the taste may not correspond to a natural, everyday beverage-drinking scenario. To elucidate the interaction between odor timing as accompanied with breath and subjective taste, further research using an olfactory device that can be used in more natural drinking conditions should be conducted.

## Conclusion

The results of the current study suggest that the retronasal odor of vanilla has an effect on the enhancement the sweet-taste perception of a gustatory stimulus. Moreover, we found that odor stimulation from the retronasal route after drinking is necessary in order for this odor-induced taste enhancement to occur. These results indicate that congruency with natural patterns involving the order of taste and odor stimuli and kinetic sensation might be a determining factor for odor-induced taste enhancement.
